# Extending the Impact of RAC1b Overexpression to Follicular Thyroid Carcinomas

**DOI:** 10.1155/2016/1972367

**Published:** 2016-04-05

**Authors:** Márcia Faria, Liliana Capinha, Joana Simões-Pereira, Maria João Bugalho, Ana Luísa Silva

**Affiliations:** ^1^Unidade de Investigação de Patobiologia Molecular, Instituto Português de Oncologia de Lisboa Francisco Gentil EPE, 1099-023 Lisboa, Portugal; ^2^Serviço de Endocrinologia, Instituto Português de Oncologia de Lisboa Francisco Gentil EPE, 1099-023 Lisboa, Portugal; ^3^Clínica Universitária de Endocrinologia, NOVA Medical School/Faculdade de Ciências Médicas, Universidade Nova de Lisboa, 1169-056 Lisboa, Portugal

## Abstract

RAC1b is a hyperactive variant of the small GTPase RAC1 known to be a relevant molecular player in different cancers. Previous studies from our group lead to the evidence that its overexpression in papillary thyroid carcinoma (PTC) is associated with an unfavorable prognosis. In the present study, we intended to extend the analysis of RAC1b expression to thyroid follicular neoplasms and to seek for clinical correlations. RAC1b expression levels were determined by RT-qPCR in thyroid follicular tumor samples comprising 23 follicular thyroid carcinomas (FTCs) and 33 follicular thyroid adenomas (FTAs). RAC1b was found to be overexpressed in 33% of carcinomas while no RAC1b overexpression was documented among follicular adenomas. Patients with a diagnosis of FTC were divided into two groups based on longitudinal evolution and final outcome. RAC1b overexpression was significantly associated with both the presence of distant metastases (*P* = 0.01) and poorer clinical outcome (*P* = 0.01) suggesting that, similarly to that previously found in PTCs, RAC1b overexpression in FTCs is also associated with worse outcomes. Furthermore, the absence of RAC1b overexpression in follicular adenomas hints its potential as a molecular marker likely to contribute, in conjunction with other putative markers, to the preoperative differential diagnosis of thyroid follicular lesions.

## 1. Introduction

More than 95% of thyroid cancers originate from thyroid follicular epithelial cells. Among the well-differentiated forms, papillary thyroid carcinoma (PTC) is the most prevalent one, accounting for 75–80% of cases, followed by the follicular thyroid carcinoma (FTC), which represents approximately 10–15% of all thyroid cancers [1]. Distinction between FTC and its benign counterpart (follicular adenoma) is impossible on cytological grounds. Pathological examination showing capsular or vascular invasion is necessary to establish the diagnosis of FTC [2–4]. Activating alterations in the canonical Ras/Raf/MEK/ERK pathway (MAPK pathway) are considered to have key role in thyroid carcinogenesis [1, 5]. The BRAF V600E activating mutation is the most frequent genetic alteration in PTCs. In FTCs, however, this alteration is virtually absent and oncogenic alterations of RAS proteins are instead the most prevalent ones [1, 5]. Although a single oncogenic alteration in MAPK pathway might be sufficient to drive thyroid cell neoplastic transformation, further supportive molecular events are likely to be associated with thyroid malignant progression leading to more aggressive phenotypes and poorer clinical outcomes. We have recently described RAC1b, a splicing variant of RAC1, as a potential new prognostic marker for clinical outcome in PTC patients [6].

RAC1 belongs to the Rho family of Ras-like small GTP-binding proteins, a class of molecular “switches” that regulate cellular functions by cycling between an inactive, GDP- and an active, GTP-bound state [7]. These small GTPases have been implicated in cancer since they regulate signaling pathways involved in processes such as gene expression, cell proliferation, and cell migration [8]. The RAC1 splice variant, RAC1b, contains 57 additional nucleotides that result in an in-frame insertion of 19 amino acid residues in the vicinity of an important regulatory region of the GTPase [9]. This confers RAC1b activating properties and a selective downstream signaling in comparison to RAC1 [10].

RAC1b overexpression has been documented in colorectal, breast, lung, and pancreatic cancer [9, 11–15]. In our previous study, we reported for the first time RAC1b expression in thyroid tissue [6]. We have, previously, shown that RAC1b is overexpressed in PTCs compared to normal thyroid tissue and that RAC1b overexpression is significantly associated with BRAF V600E mutation and poorer clinical outcome in PTC [6]. Here, we aimed to broaden the study of RAC1b expression to follicular lesions including follicular adenomas and follicular carcinomas and to seek for clinical correlations.

## 2. Materials and Methods

### 2.1. Tumor Samples

Samples representative of 23 FTCs and samples representative of 33 follicular adenomas from 56 patients, who underwent surgery at our institution, were analyzed. Samples were collected at surgery and immediately frozen and stored in liquid nitrogen. Tissue sample collection was carried out in accordance with protocols approved by the institutional review board and written informed consent was obtained together with the consent for surgery.

### 2.2. RNA Extraction, cDNA Synthesis, and Mutational Screening of* KRAS*,* HRAS*, and* NRAS*

Total RNA was obtained from frozen tissues using RNA easykit (Qiagen), according to manufacturer's instructions, and 2 *μ*g was reverse transcribed using random primers and SuperScript II (Invitrogen).

Mutational analysis of* KRAS, HRAS*, and* NRAS* was performed by Sanger sequencing method: the full coding regions of* RAS* transcripts were amplified by reverse transcription polymerase chain reaction (RT-PCR) (primers and PCR conditions available upon request). PCR purified products were directly sequenced using BigDye® Terminator v1.1 Cycle Sequencing Kit (Applied Biosystems, Foster City, CA, USA). Sequencing products were analyzed using ABI PRISM 3130 Genetic Analyzer (Applied Biosystems).

### 2.3. RT-qPCR

The RAC1b and total RAC1 expression levels were quantified by quantitative reverse transcription polymerase chain reaction (RT-qPCR) on an ABI Prism 7900HT Sequence Detection System, as previously described [6]. For each sample, RAC1b levels were normalized to total RAC1 (RAC1b + RAC1) expression level. RAC1b normalized values were then expressed relative to that of a pool of normal thyroid tissues, used as reference sample. Expression values correspond to arbitrary units representing fold differences relative to the reference sample. RAC1b overexpression was defined as a value above a threshold level of RAC1b expression corresponding to the mean plus two standard deviations of the RAC1b expression level found in the set of normal thyroid tissue samples; this threshold level was set at 2.133 (arbitrary units). Similarly, to monitor total RAC1 expression among samples, total Rac1 levels were normalized to beta-actin expression level (housekeeping gene normalization) and expressed relative to that of the reference sample.

### 2.4. Protein Lysates and Western Blotting

Total protein lysates were prepared from frozen thyroid tissues as previously described [6]. The primary antibodies rabbit polyclonal anti-Rac1b (Millipore) and mouse monoclonal anti-*β*-actin (Sigma) were used in Western blot at 1 : 1000 and 1 : 3000 dilutions, respectively. Detection was carried out using secondary peroxidase-conjugated anti-mouse IgG (Bio-Rad) or anti-rabbit IgG (Bio-Rad) antibodies followed by chemiluminescence.

### 2.5. Statistical Analysis

Statistical analysis was carried out using GraphPad Prism statistical software (San Diego, CA). When appropriate, values are expressed as mean ± SD. Statistical comparisons of rates and proportions were made using unpaired two-tailed Student's *t*-test or the two-tailed Fisher exact test, when appropriated. Statistical significance was accepted at *P* < 0.05.

## 3. Results

### 3.1. RAC1b Is Overexpressed in FTC

**Figure 1:**
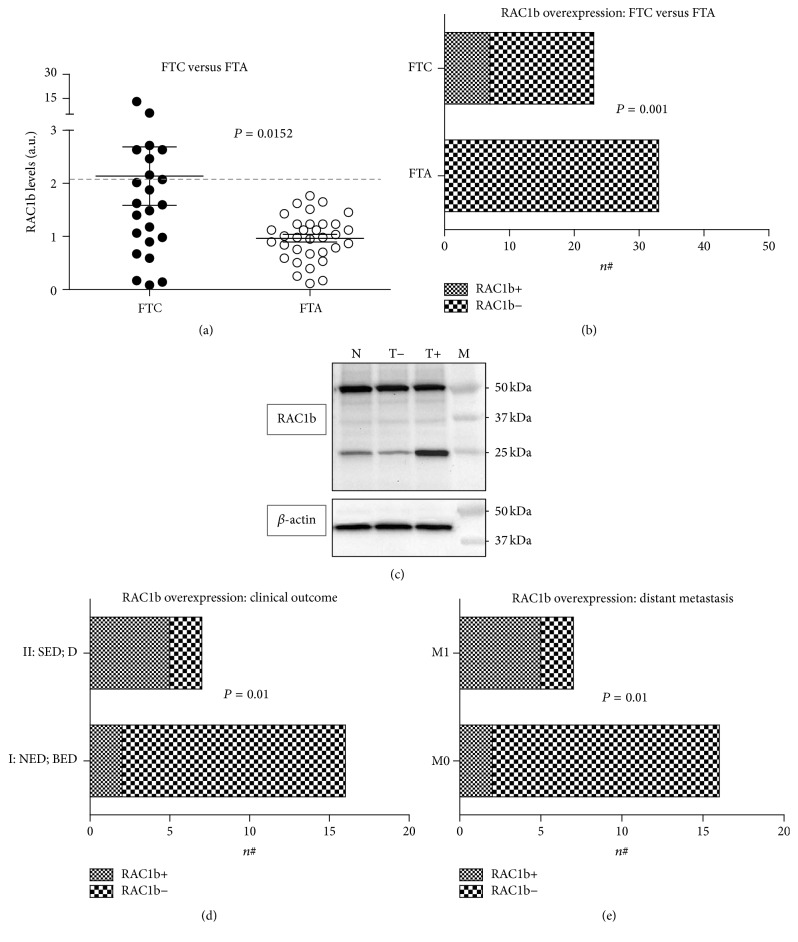
RAC1b expression in follicular thyroid tumors. (a) Expression levels of RAC1b among FTCs (*n* = 23) and FTAs (*n* = 33). RAC1b expression levels, quantified by qRT-PCR, correspond to arbitrary units representing fold differences relative to the reference sample; the threshold value defining RAC1b overexpression was set at 2.133 (corresponding to the mean plus two standard deviations of RAC1b expression level in a set of normal thyroid tissue samples). (b) Comparative analysis of RAC1b overexpression (RAC1b+) between follicular thyroid carcinomas (FTC) and follicular thyroid adenomas (FTA). (c) Western blot analysis of RAC1b protein levels in normal thyroid (N) and RAC1b overexpressing (T+) and nonoverexpressing (T−) tumors; protein molecular weight marker (M). (d) Association of RAC1b overexpression (RAC1b+) with clinical outcome (Group I: NED: no evidence of disease, BED: biochemical evidence of disease; Group II: SED: structural evidence of disease, D: death due to disease). (e) Association of RAC1b overexpression (RAC1b+) with the presence (M1) or absence (M0) of distant metastases.

Total RAC1 and RAC1b expression levels were assessed by RT-qPCR. No significant differences in total RAC1 levels were found between FTCs and FTAs samples. RAC1b expression levels in tumor samples were obtained following normalization to the levels from a pool of normal thyroid tissues used as reference sample in all RT-qPCR assays. The mean level for RAC1b expression was significantly higher in FTCs (2.137 ± 0.5515) compared to FTAs (0.9656 ± 0.07342) (*P* value = 0.0152, two-tailed Student's *t*-test), corresponding to a 2.2 mean fold increase in the levels of RAC1b expression (Figure 1(a)).

In order to distinguish tumors that overexpressed RAC1b from those that did not, we defined a threshold level of RAC1b expression relative to the normal thyroid tissue (see Section 2) above where we considered RAC1b to be overexpressed. RAC1b was found to be overexpressed in 7 out of 23 FTCs (30%). Notably, none of the cases belonging to the FTAs group was found to overexpress RAC1b. Consistently, the difference in RAC1b overexpression between follicular adenomas and carcinomas was statistically significant (*P* value = 0.001, two-tailed Fisher's exact test; Figure 1(b)).

Since the assessment of RAC1b overexpression by immunohistochemistry in paraffin-embedded tissues might be relevant for diagnostic purposes, we further explored this possibility using the only RAC1b specific antibody that is commercially available. Unfortunately, we were not able to detect a clear difference in RAC1b protein levels either comparing RAC1b overexpressing and nonoverexpressing tumors or tumor and normal tissue. Nevertheless, a notorious difference in RAC1b expression was observed when protein levels were assessed by Western blot. This discrepancy is likely to be due to antibody nonspecific binding as pointed out by the band of *≅*50 kDa observed in the immunoblot (Figure 1(c)) that hampers an accurate analysis by immunohistochemistry.

### 3.2. RAC1b Overexpression Is Associated with Presence of Distant Metastases and Poorer Clinical Outcomes

To investigate whether RAC1b overexpression was associated with histopathological parameters and clinical outcome, FTCs were grouped based on the clinical data available (see Table 1).

A comparative analysis between FTC patients without distant metastases (M0, 16 cases) versus FTC patients with distant metastases (M1, 7 cases) disclosed a higher prevalence of RAC1b overexpression in the latter group (M1—5/7; M0—2/16). The association between RAC1b overexpression and presence of distant metastases was statistically significant (*P* value = 0.01; Figure 1(c)). No statistical significant correlation was found between RAC1b overexpression and histopathological features such as multifocality, angioinvasion, Hürthle cell subtype, or presence of poorly differentiated areas.

FTCs were grouped based on the final clinical outcome. Group I (70% of patients) included patients who reached full sustained remission (no evidence of disease) and patients presenting only biochemical evidence of disease. In contrast, Group II (30% of patients) included patients with structural evidence of disease and those who died due to disease progression. The correlation between RAC1b overexpression and Group II was statistically significant (*P* value = 0.01; Figure 1(d)), reinforcing that RAC1b might be associated with poorer clinical outcomes in FTC patients.

## 4. Discussion

At present, stratification risk of patients with follicular cell-derived thyroid carcinomas mainly relies on clinical and histological criteria proved to be insufficient to tailor case management to individual risk levels [16]. A major effort to improve the panel of prognostic indicators has entailed the molecular characterization of thyroid tumors, seeking for molecular markers with a reliable prognostic value [17, 18].

Previous studies from our group provided evidence for a contributory role of RAC1b in PTC development and clinical outcome [6]. We, therefore, hypothesized that RAC1b might also be involved in follicular thyroid carcinoma development. Moreover, taking into account the impossibility of distinguishing a follicular carcinoma from a follicular adenoma, preoperatively, we wondered whether the RAC1b expression could contribute to the distinction of these two entities.

Our data suggest that RAC1b is likely to have a differential expression in follicular adenomas and carcinomas, since RAC1b was overexpressed in 30% of FTCs whereas no overexpression was found among FTAs. Furthermore, we also found a significant correlation between RAC1b overexpression and the presence of distant metastases, hence, with poorer outcome.

Papillary thyroid carcinoma and follicular thyroid carcinoma are collectively designated well-differentiated thyroid carcinomas (DTCs). Nonetheless, the underlying genetic alterations promoting the development of these two types of thyroid cancer are different. The BRAF V600E mutation occurs in approximately 45% of PTCs. It is not definitely established whether BRAF V600E initiates PTC tumorigenesis or is a secondary event. In a previous study, we have shown that RAC1b overexpression significantly associates with BRAF V600E mutation in PTCs [10]. This suggests that a functional cooperation between RAC1b and BRAF V600E may play a role in PTC progression, similarly to that previously described in colorectal cancer [11]. In fact, in colorectal tumors, the elevated levels of Rac1b expression were significantly associated with mutant BRAF but not with mutant K-RAS genotype, suggesting that RAC1b is needed to sustain BRAF-induced but not RAS-induced cell transformation. The mechanism of action of RAC1b in tumorigenesis may, however, differ in other tissues.

Concerning follicular thyroid lesions, BRAF V600E is virtually absent in this tumor subtype and the most common MAPK-pathway alteration is the presence of RAS mutations. Notably and contrary to that found in colorectal cancer, oncogenic alterations in K-RAS have also been associated with RAC1b overexpression in lung cancer and RAC1b was shown to promote K-RAS-induced lung tumorigenesis [13]. In our series of FTC patients no association between RAC1b overexpression and RAS mutations could be observed. However, this might be due to the small sample size. Despite these, the incidence of RAS mutations in follicular adenomas, which are considered premalignant lesions, suggests a role for RAS in early phases of follicular cell tumorigenesis. This is in agreement with the hypothesis that the progression of a premalignant lesion into cancer, with different grades of aggressiveness, is linked to a continuum of molecular changes dependent on late and/or modifier events that are not necessarily exclusive of a specific type of tumor or mechanism of tumorigenesis. Overexpression of RAC1b may thus be one of these modifier events that can occur in different subtypes of DTCs despite having different triggers.

## 5. Conclusion

The clinical usefulness of RAC1b overexpression in the context of thyroid malignancies remains to be definitively established. Nevertheless, our data suggest that RAC1b might be involved in the modulation of malignant progression of FTCs, contributing to poorer clinical outcomes, similarly to that previously documented in PTCs. Our data also support the potential of RAC1b as a molecular marker likely to contribute, in conjunction with other putative markers, to the diagnosis and prognosis of FTCs.

## Figures and Tables

**Table 1:** Clinical data: ID, patient identification; M, male; F, female; HCC, Hürthle cells carcinoma; FTC, follicular thyroid carcinoma; TNM, tumor, node, and metastases staging; WI, widely invasive; EI, extrathyroidal invasion; M, multifocal; A, angioinvasion; PD, poorly differentiated areas; ^131^I, number of ^131^I treatments; TT, total thyroidectomy; HT, hemithyroidectomy; TTC, total thyroidectomy with cervical dissection; TG, thyroglobulin; M1, distant metastases; n.t., not tested; NED, no evidence of disease; BED, biochemical evidence of disease; SED, structural evidence of disease; D; death due to disease; I, remission or biochemical disease; II, structural disease or death due to disease.

Patient			Histopathology			Treatment	Follow-up				Clinical outcome					Molecular analysis	
ID	Gender	Age at diagnosis (years)	TNM	Pattern	WI/EI/M/A/PD	Surgery/^131^I	Years	Last TG (ng/mL)	Anti-TG	M1	NED	BED	SED	D	Group	RAS (K/N/H)	RAC1b overexpression
1	F	68	T2NxMx	HCC	(−/−/−/+/−)	TT/1	5	<0.2	0	—	✓				I	(—/—/—)	−
2	F	45	T3NxMx	HCC	(−/−/−/+/−)	HT/0	8	1.7	n.t.	—	✓				I	(—/—/—)	−
3	F	40	T3NxMx	FTC	(−/−/−/+/−)	HT/0	7	1.9	n.t.	—	✓				I	(—/Q61R/—)	−
4	M	38	T3NxMx	FTC	(−/−/−/−/−)	TT/1	13	<0.2	0	—	✓				I	(—/—/—)	−
5	F	70	T3NxMx	FTC	(−/−/−/+/−)	TT/2	12	<0.2	0	—	✓				I	(—/—/—)	−
6	F	63	T3NxMx	FTC	(−/−/−/+/−)	TT/1	7	<0.2	0	—	✓				I	(—/—/—)	−
7	F	57	T2NxMx	HCC	(−/−/−/−/−)	HT/0	16	8.7	n.t.	—	✓				I	(—/—/—)	+
8	F	59	T2NxMx	FTC	(−/−/−/−/−)	TT/1	15	<0.2	0	—	✓				I	(—/—/—)	+
9	M	38	T2NxMx	FTC	(−/−/−/−/−)	TT/1	11	<0.2	0	—	✓				I	(—/—/—)	−
10	F	39	T3NxMx	FTC	(+/−/−/+/+)	TT/2	9	<0.2	0	—	✓				I	(—/—/—)	−
11	F	34	T2NxMx	HCC	(−/−/−/−/−)	HT/0	7	11.1	n.t.	—	✓				I	(—/—/—)	−
12	F	39	T2NxMx	FTC	(−/−/−/−/−)	TT/2	7	2.7	n.t.	—		✓			I	(—/—/—)	−
13	F	81	T3NxMx	HCC	(−/+/−/−/−)	TT/1	4	<0.2	0	—	✓				I	(—/—/n.t.)	−
14	M	39	T3NxMx	FTC	(−/−/−/−/−)	TT/1	7	<0.2	0	—	✓				I	(—/—/—)	−
15	F	49	TxNxMx	FTC	(−/−/−/+/−)	HT/0	23	<0.2	0	—	✓				I	n.t.	−
16	F	65	T2NxMx	FTC	(−/−/−/+/−)	HT/0	6	<0.2	0	—	✓				I	(—/—/—)	−
17	F	71	T1NxM1	HCC	(+/+/+/+/−)	TT/2	4	300000	0	Lung adrenal gland				✓	II	n.t.	−
18	F	42	T3NxMx	HCC	(−/−/−/+/+)	TT/6	10	2108	0	Lung				✓	II	(—/—/—)	+
19	M	58	T3NxM1	FTC	(−/−/−/+/−)	TT/4	0	376	0	Bone			✓		II	(—/Q61R/—)	+
20	M	53	T2NxMx	HCC	(+/−/+/+/−)	TT/4	7	632	0	Lung				✓	II	(—/—/—)	+
21	M	69	T2NxM1	FTC	(−/−/−/+/−)	TT/5	2	4668	0	Bone, inguinal node				✓	II	(—/—/—)	+
22	M	64	T4aN1bMx	FTC	(+/+/−/+/+)	TTC/3	2	28.6	0	Lung			✓		II	(—/—/—)	+
23	F	87	T3NxMx	FTC	(+/+/−/+/−)	TT/5	10	43500	n.t.	Soft tissues			✓		II	(—/Q61L/—)	−

## References

[1] Bhaijee F., Nikiforov Y. E. (2011). Molecular analysis of thyroid tumors. *Endocrine Pathology*.

[2] Cooper D. S., Doherty G. M., Haugen B. R. (2006). Management guidelines for patients with thyroid nodules and differentiated thyroid cancer. *Thyroid*.

[3] Haugen B. R., Alexander E. K., Bible K. C. (2016). 2015 American Thyroid Association Management Guidelines for Adult Patients with Thyroid Nodules and Differentiated Thyroid Cancer: the American Thyroid Association Guidelines Task Force on Thyroid Nodules and Differentiated Thyroid Cancer. *Thyroid*.

[4] DeLellis R. A. (2006). Pathology and genetics of thyroid carcinoma. *Journal of Surgical Oncology*.

[5] Romitti M., Ceolin L., Siqueira D. R., Ferreira C. V., Wajner S. M., Maia A. L. (2013). Signaling pathways in follicular cell-derived thyroid carcinomas (review). *International Journal of Oncology*.

[6] Silva A. L., Carmo F., Bugalho M. J. (2013). RAC1b overexpression in papillary thyroid carcinoma: a role to unravel. *European Journal of Endocrinology*.

[7] Jaffe A. B., Hall A. (2005). Rho GTPases: biochemistry and biology. *Annual Review of Cell and Developmental Biology*.

[8] Orgaz J. L., Herraiz C., Sanz-Moreno V. (2014). Rho GTPases modulate malignant transformation of tumor cells. *Small GTPases*.

[9] Jordan P., Brazão R., Boavida M. G., Gespach C., Chastre E. (1999). Cloning of a novel human Rac1b splice variant with increased expression in colorectal tumors. *Oncogene*.

[10] Fiegen D., Haeusler L.-C., Blumenstein L. (2004). Alternative Splicing of Rac1 Generates Rac1b, a Self-activating GTPase. *Journal of Biological Chemistry*.

[11] Matos P., Oliveira C., Velho S. (2008). B-RafV600E cooperates with alternative spliced Rac1b to sustain colorectal cancer cell survival. *Gastroenterology*.

[12] Schnelzer A., Prechtel D., Knaus U. (2000). Rac1 in human breast cancer: overexpression, mutation analysis, and characterization of a new isoform, Rac1b. *Oncogene*.

[13] Zhou C., Licciulli S., Avila J. L. (2013). The Rac1 splice form Rac1b promotes K-ras-induced lung tumorigenesis. *Oncogene*.

[14] Mehner C., Miller E., Khauv D. (2014). Tumor cell-derived MMP3 orchestrates Rac1b and tissue alterations that promote pancreatic adenocarcinoma. *Molecular Cancer Research*.

[15] Ungefroren H., Sebens S., Giehl K. (2014). Rac1b negatively regulates TGF-*β*1-induced cell motility in pancreatic ductal epithelial cells by suppressing Smad signalling. *Oncotarget*.

[16] Sipos J. A., Mazzaferri E. L. (2010). Thyroid cancer epidemiology and prognostic variables. *Clinical Oncology*.

[17] Witt R. L., Ferris R. L., Pribitkin E. A., Sherman S. I., Steward D. L., Nikiforov Y. E. (2013). Diagnosis and management of differentiated thyroid cancer using molecular biology. *The Laryngoscope*.

[18] Nagar S., Ahmed S., Peeples C. (2014). Evaluation of genetic biomarkers for distinguishing benign from malignant thyroid neoplasms. *American Journal of Surgery*.

